# Supplementary material for: Increasing aridity threatens the sexual regeneration of Quercus ilex(holm oak) in Mediterranean ecosystems
Source: PLoS One. 2020 Oct 14;15(10):e0239755. doi: 10.1371/journal.pone.0239755 (PMC7556486; doi:10.1371/journal.pone.0239755)
Supplement: S1 Table — MAP = Mean Annual Precipitation, PET = Potential Evapotranspiration and MAT = Mean Annual Temperature. Plot identification codes follow Moreno-de-las-Heras et al. 2018. Ecosystems, 21: 1295–1305. (DOCX) [file pone.0239755.s001.docx]

**S1 Table. Geographical and environmental characteristics of the study plots.**

| Precipitation level | Locality | Plot | Geographic coordinates | | Altitude  (m.a.s.l.) | Slope  (°) | MAP^1^  (mm) | PET^2^  (mm) | MAT^3^  (°) |
| --- | --- | --- | --- | --- | --- | --- | --- | --- | --- |
|  |  |  | **N** | **W** |  |  |  |  |  |
| Semi-arid | Cella | S4 | 40° 29’ 18.3’’ | 1° 25’ 34.0’’ | 1257 | 5.6 | 425 | 923 | 10.6 |
| Semi-arid | Villafranca del Campo | S6 | 40° 39’ 18.4’’ | 1° 24’ 30.6’’ | 1175 | 4.3 | 444 | 926 | 10.7 |
| Semi-arid | Villafranca del Campo | S8 | 40° 39’ 18.0’’ | 1° 24’ 10.8’’ | 1195 | 5.0 | 449 | 926 | 10.7 |
| Semi-arid | San Blas | S44 | 40° 19’ 27.7’’ | 1° 11’ 43.6’’ | 1070 | 2.5 | 431 | 933 | 12.0 |
| Semi-arid | San Blas | S42 | 40° 20’ 06.0’’ | 1° 12’ 31.7’’ | 1077 | 5.6 | 444 | 932 | 11.8 |
| Semi-arid | Santa Eulalia | S13 | 40° 32’ 53.8’’ | 1° 22’ 41.5’’ | 1162 | 4.9 | 425 | 923 | 10.6 |
| Semi-arid | Cella | S50 | 40° 33’ 01.6’’ | 1° 23’ 01.0’’ | 1191 | 3.8 | 443 | 940 | 10.5 |
| Semi-arid | Santa Eulalia | S22 | 40° 32’ 07.8’’ | 1° 21’ 33.7’’ | 1112 | 4.2 | 450 | 929 | 10.8 |
| Semi-arid | Santa Eulalia | S20 | 40° 32’ 22.9’’ | 1° 21’ 43.2’’ | 1121 | 4.0 | 435 | 919 | 10.8 |
| Semi-arid | Santa Eulalia | S25 | 40° 33’ 30.2’’ | 1° 21’ 21.8’’ | 1119 | 2.1 | 424 | 926 | 10.7 |
| Semi-arid | Santa Eulalia | S27 | 40° 33’ 15.1’’ | 1° 21’ 12.4’’ | 1109 | 4.3 | 434 | 929 | 10.7 |
| Sub-humid | Traid | S108 | 40° 40’ 42.8’’ | 1° 47’ 30.0’’ | 1399 | 4.8 | 647 | 932 | 9.8 |
| Sub-humid | Vilafranca | S123 | 40° 25’ 08.5’’ | 0° 16’ 32.1’’ | 1211 | 4.8 | 630 | 876 | 10.7 |
| Sub-humid | Traid | S115 | 40° 40’ 19.2’’ | 1° 42’ 51.8’’ | 1394 | 4.9 | 643 | 930 | 9.8 |
| Sub-humid | Vilafranca | S127 | 40° 23’ 52.8’’ | 0° 16’ 05.7’’ | 1196 | 3.3 | 613 | 882 | 10.9 |
| Sub-humid | Vilafranca | S138 | 40° 27’ 47.1’’ | 0° 12’ 19.7’’ | 1253 | 2.0 | 646 | 871 | 11.1 |
| Sub-humid | Vilafranca | S133 | 40° 24’ 29.6’’ | 0° 15’ 34.7’’ | 1160 | 1.5 | 640 | 885 | 11.0 |

Legend: ^1^MAP= Mean Annual Precipitation, ^2^PET= Potential Evapotranspiration and ^3^MAT= Mean Annual Temperature. Plot identification codes follow Moreno-de-las-Heras et al. Aridity induces nonlinear effects of human disturbance on Precipitation-Use Efficiency of Iberian woodlands. Ecosystems; 2018; 21: 1295-1305. https://doi.org/10.1007/s10021-017-0219-8
