# Supplementary material for: Increasing aridity threatens the sexual regeneration of Quercus ilex(holm oak) in Mediterranean ecosystems
Source: PLoS One. 2020 Oct 14;15(10):e0239755. doi: 10.1371/journal.pone.0239755 (PMC7556486; doi:10.1371/journal.pone.0239755)
Supplement: S2 Table — Bold letters indicate that we found association of that species with recruits of Q. ilex in our study area. Life form: T = tree, LS = large size shrub, MS = medium size shrub, SS = small size shrub and PS = prostrate shrub. Precipitation level: SA = semi-arid, SH = sub-humid, B = both. (*) Several species of the sectio Rosa caninae. (DOCX) [file pone.0239755.s002.docx]

**S2 Table. List of plant species with potential to act as nurse for *Quercus ilex* recruits in our study area.**

Species Life form Aridity

*Arctostaphylos uva-ursi* (L.) Spreng. PS SH

*Bupleurum fruticescens* SS SA

*Buxus sempervirens* L. LS SH

*Erinacea anthyllis* Link SS SH

*Genista pumila* (Debeaux & É. Rev. ex Hervier) Vierh. SS B

*Genista scorpius* (L.) DC. SS B

***Juniperus communis*** L. LS SH

***Juniperus phoenicea*** L. LS B

***Juniperus sabina*** L. PS SH

***Juniperus thurifera*** L. T B

*Lavandula latifolia* Medik. SS B

***Pinus nigra*** J. F. Arnold T SH

***Quercus faginea*** Lam. T SH

***Quercus ilex*** L. T B

*Rhamnus saxatilis* Jacq. MS B

*Rosa** MS SH

*Salvia lavandulifolia* Vahl SS B

Nomenclature follows Castroviejo, S. (coord.) 1986-2012. Flora Ibérica, vols 1-18, 20-21. Real Jardín Botánico, CSIC, Madrid). Bold letters indicate that we found association of that species with recruits of *Q. ilex* in our study area. Life form: T =tree, LS = large size shrub, MS = medium size shrub, SS = small size shrub and PS = prostrate shrub. Precipitation level: SA = semi-arid, SH = sub-humid, B = both.

(*) Several species of the *sectio Rosa caninae*
