# Supplementary material for: Increasing aridity threatens the sexual regeneration of Quercus ilex(holm oak) in Mediterranean ecosystems
Source: PLoS One. 2020 Oct 14;15(10):e0239755. doi: 10.1371/journal.pone.0239755 (PMC7556486; doi:10.1371/journal.pone.0239755)
Supplement: S3 Table — We consider saplings those recruits aged 15 and younger, and juveniles those aged 16 to 50 years. The values of intensity of past deforestation, area occupied by reproductive Quercus ilex and nurses, soil availability and herbivory pressure are ratios. The intensity of past deforestation indicates the loss of tree cover relative to the maximum tree cover in each precipitation level. The area of reproductive Q. ilex and nurse plants is the proportion of the area of the plot covered by the canopy of those species. The availability of soil is the proportion of points in the plot where an acorn could germinate and take root. Herbivore pressure is the proportion of subplots in each plot that showed signs of the presence of domestic and wild herbivores (See methods for a detailed variable description). (DOCX) [file pone.0239755.s003.docx]

**S3 Table. Summary of the measured variables in each plot.**

| Precipitation level | Plot | Past deforestation Intensity Index | Relative cover of reproductive *Quercus ilex* | Relative cover of nurse plants | Soil availability | Herbivore pressure | Number of *Quercus ilex* recruits | | Number of *Quercus ilex* recruits associated with nurse plants | | |
| --- | --- | --- | --- | --- | --- | --- | --- | --- | --- | --- | --- |
|  |  |  |  |  |  |  | **Saplings** | **Juveniles** | **Controls** | **Saplings** | **Juveniles** |
| Semi-arid | S4 | 0.0000 | 0.4073 | 0.4799 | 0.7350 | 0.8372 | 3 | 31 | 37 | 3 | 23 |
| Semi-arid | S6 | 0.1656 | 0.3859 | 0.4012 | 0.6850 | 0.8261 | 1 | 11 | 27 | 1 | 3 |
| Semi-arid | S8 | 0.1902 | 0.2593 | 0.2839 | 0.6400 | 0.8696 | 0 | 11 | 18 | 0 | 2 |
| Semi-arid | S44 | 0.2221 | 0.3504 | 0.5328 | 0.7500 | 0.3696 | 8 | 23 | 28 | 8 | 12 |
| Semi-arid | S42 | 0.3288 | 0.2187 | 0.4197 | 0.7600 | 0.8043 | 3 | 28 | 46 | 3 | 13 |
| Semi-arid | S13 | 0.3962 | 0.1270 | 0.1390 | 0.6650 | 0.9348 | 0 | 29 | 20 | 0 | 1 |
| Semi-arid | S50 | 0.4574 | 0.1258 | 0.2957 | 0.7950 | 0.7609 | 4 | 2 | 21 | 4 | 2 |
| Semi-arid | S22 | 0.6268 | 0.2390 | 0.2427 | 0.4450 | 0.9565 | 1 | 2 | 5 | 1 | 0 |
| Semi-arid | S20 | 0.6858 | 0.1560 | 0.1655 | 0.5900 | 0.9783 | 1 | 3 | 14 | 1 | 1 |
| Semi-arid | S25 | 0.7451 | 0.1215 | 0.1332 | 0.6450 | 0.7609 | 0 | 3 | 13 | 0 | 0 |
| Semi-arid | S27 | 0.9293 | 0.0235 | 0.0470 | 0.6650 | 0.9565 | 1 | 16 | 10 | 1 | 2 |
| Sub-humid | S108 | 0.3098 | 0.4392 | 0.6004 | 0.6200 | 0.9783 | 70 | 98 | 47 | 68 | 81 |
| Sub-humid | S123 | 0.3661 | 0.3014 | 0.6057 | 0.6450 | 0.8043 | 374 | 256 | 59 | 362 | 232 |
| Sub-humid | S115 | 0.4347 | 0.1850 | 0.4422 | 0.7750 | 1.0000 | 9 | 47 | 54 | 6 | 43 |
| Sub-humid | S127 | 0.5424 | 0.1084 | 0.5590 | 0.7050 | 0.2826 | 91 | 422 | 59 | 84 | 419 |
| Sub-humid | S138 | 0.7957 | 0.0238 | 0.3219 | 0.5750 | 0.8913 | 48 | 100 | 54 | 46 | 76 |
| Sub-humid | S133 | 0.9168 | 0.0019 | 0.2421 | 0.2900 | 0.7174 | 0 | 1 | 33 | 0 | 0 |

Legend: We consider saplings those recruits aged 15 and younger, and juveniles those aged 16 to 50 years. The values of intensity of past deforestation, area occupied by reproductive *Quercus ilex* and nurses, soil availability and herbivory pressure are ratios. The intensity of past deforestation indicates the loss of tree cover relative to the maximum tree cover in each precipitation level. The area of reproductive *Q. ilex* and nurse plants is the proportion of the area of the plot covered by the canopy of those species. The availability of soil is the proportion of points in the plot where an acorn could germinate and take root. Herbivore pressure is the proportion of subplots in each plot that showed signs of the presence of domestic and wild herbivores (See methods for a detailed variable description).
