# Supplementary material for: Increasing aridity threatens the sexual regeneration of Quercus ilex(holm oak) in Mediterranean ecosystems
Source: PLoS One. 2020 Oct 14;15(10):e0239755. doi: 10.1371/journal.pone.0239755 (PMC7556486; doi:10.1371/journal.pone.0239755)
Supplement: S1 Appendix — (DOCX) [file pone.0239755.s004.docx]

**S1 Appendix. Comparison of the abundance of herbivores at the study sites during the last century and at the time of sampling.**

The populations of domestic and large wild herbivores can exert great pressure on the recruitment of *Q. ilex*. Therefore, we examined the indicators of herbivore pressure to ensure that they remained homogeneous in both precipitation levels and along the gradient of past deforestation intensity during the 50 years prior to this study.

Data sources and statistical methods:

To estimate past herbivore pressure, we obtained data of livestock density (sheep, goats, cattle, horses, donkeys and mules) and wild herbivores (*Cervus elaphus* L., *Capreolus capreolus* L., *Sus scrofa* L. and *Capra pyrenaica* Schinz) for each province where the sampling plots were located. We obtained the data on number of domestic herbivores and surface available for grazing from the Spanish agrarian censuses of 1865, 1920, 1962, 1982 and 1999 (Instituto Nacional de Estadística, [http://www.ine.es](http://www.ine.es/en/welcome.shtml), last accessed 01/02/2020). Because equivalent data on wild herbivore numbers are not available, we estimated density values for 1948, 1998 and 2010 from the published literature (Gortázar et al., 2000; Marco et al., 2011).

To estimate current herbivore pressure, we estimated the probability of finding evidence of domestic and wild herbivore use in the plots. We systematically sampled for evidence of herbivore presence, mostly faecal pellets on the ground, but also footprints, wool and hairs attached to shrubs and trees, in the forty-six 10 x 14 m sections per plot. Because of the dry Mediterranean climatic conditions in our study area, we assumed that the rate of degradation of faecal pellets is slow enough to allow estimation of the presence of herbivores during the last four years (2014 to 2017) (Hibert et al., 2011; Vinograd et al., 2019). We fitted generalized binomial logistic models to the proportion of the 46 sections per plot with evidence of the presence of herbivores. We used precipitation level, past deforestation intensity, and their interaction as fixed effects, and locality as random effect because the 17 plots where clustered in municipalities.

Results and discussion:

Herbivore pressure in the past by livestock was similar in the three provinces where our study plots were located. It steadily declined at a similar rate until now (Table S2.1). In the period 1962-1982, when the oldest recruits in our study germinated, cattle density was approximately half that of 1865, but it was 10 to 20 times lower in 1999, when the youngest recruits germinated.

In 1848, all wild herbivores species (*Cervus elaphus* L., *Capreolus capreolus* L., *Sus scrofa* L. and *Capra pyrenaica* Schinz) were absent in our study localities (Gortázar et al. 2000). In the 1960s, a few individuals of *C. elaphus* were introduced in an area near the plots in the Guadalajara province, from where their populations expanded, slowly over the next decades and faster from the end of the 20th century to present. From 1960 onwards, the decrease of the rural population accelerated enormously, resulting in the spontaneous recolonization by *S. scrofa* and *C. capreolus* in all sites (Gortázar et al., 2000) and by *C. pyrenaica* in Vilafranca (Castelló province). In the 2010s, the density of wild herbivores in the studied areas was between 0.10 and 0.20 individuals ha^-1^ (Marco et al., 2011; Prada et al., 2013), a value comparable to that of livestock density in 1999 (Table S2.1).

The model on the estimated current herbivore pressure in out plots showed that the proportion of sections of the plots with evidence of presence of herbivores was constant regarding precipitation level, intensity of past deforestation and their interaction (Table S2.2). These results did not change when we removed the effect of the interaction (results not shown). Although the spatial scale of the data used on the historical presence of livestock and wild herbivores exceeds that at the plot level, the similarity of the values obtained and their temporal dynamics make it reasonable to assume that the intensity of herbivory in the periods of recruitment considered in this study could not be very different between plots.

Table S2.1 Livestock density (individual ha^-1^) in the provinces where the sampling plots were located. Data obtained from the Spanish Statistical Office: https://www.ine.es/en/.

Province/Year 1865 1920 1962 1982 1999

Castelló 1.06 0.71 0.69 0.46 0.08

Guadalajara 1.65 1.57 1.04 0.57 0.07

Teruel 1.24 0.85 0.77 0.85 0.12

Table S2.2 Model summary of GLMM on the probability of founding evidences of the presence of herbivores, domestic and wild combined, in the same sections of the plots used for recruitment sampling.

Estimate Std. Error p-value

(Intercept) 1.4214 0.6671 0.0331

Precipitation level (Sub-humid) 0.3563 1.1840 0.7635

Deforestation intensity 0.1637 0.8322 0.8441

Precipitation level: Deforestation 0.8917 1.1246 0.4279

References:

Gortázar, C., Herrero, J., Villafuerte, R., Marco, J., 2000. Historical examination of the status of large mammals in Aragon, Spain. Mammalia, 64(4), 411-422. https://doi.org/10.1515/mamm.2000.64.4.411

Hibert, F., Maillard, D., Fritz, H., Garel, M., Abdou, H. N., Winterton, P., 2011. Ageing of ungulate pellets in semi-arid landscapes: how the shade of colour can refine pellet-group counts. European Journal of Wildlife Research, 57(3), 495-503. https://doi.org/10.1007/s10344-010-0458-x

Marco, J., Herrero, J., Escudero, M. A., Fernández-Arberas, O., Ferreres, J, García-Serrano, A., Giménez-Anaya, A., Labrata, J. L., Monrabal, L. Prada, C., 2011. Veinte años de seguimiento poblacional de ungulados silvetres en Aragón. Pirineos, 166, 135-153. https://doi.org/10.3989/pirineos.2011.166007

Vinograd, A., Zaady, E., Kigel, J., 2019. Dynamics of soil nutrients in abandoned sheep corrals in semi-arid Mediterranean planted forests under grazing. Journal of Arid Environments, 164, 38-45. https://doi.org/10.1016/j.jaridenv.2019.02.007

Prada, C., García-Serrano, A., Arteaga, Z., Fernández-Arberas, O., Herrero, J., 2013. Seguimiento de los ungulados silvestres en Castellón durante 2012. Technical report for the “Servei de Vida Silvestre de la Comunitat Valenciana”.
