# Supplementary material for: Increasing aridity threatens the sexual regeneration of Quercus ilex(holm oak) in Mediterranean ecosystems
Source: PLoS One. 2020 Oct 14;15(10):e0239755. doi: 10.1371/journal.pone.0239755 (PMC7556486; doi:10.1371/journal.pone.0239755)
Supplement: S2 Appendix — (DOCX) [file pone.0239755.s005.docx]

**S2 Appendix. Modelling the relationships between the estimated age from ring counts and root collar diameter in the field of *Quercus ilex* recruits.**

Objective:

Develop a model that relates the diameter of the root collar of recruits measured in the field to its age determined from the number of tree rings.

Material and methods:

We dug 110 and 61 recruits from the semi-arid and sub-humid *Quercus ilex* plots, respectively, covering the entire range of root-collar diameters up to 50 mm in each precipitation level, and submitted them for plant age determination to the Botany Laboratory of the University of Valladolid in Soria. At the laboratory, anatomical cross-sections of 10 μm thickness were produced with a sledge microtome (Gärtner et al., 2015). These cross-sections were then placed on a slide and stained with Alcian blue (1% solution in acetic acid) and safranin (1% solution in ethanol). Afterwards, the cross-sections were dehydrated using a series of ethanol solutions of increasing concentration, washed with xylol, and permanently preserved by embedding them into Eukitt glue. Annual rings were counted in slides with a Nikon Eclipse 50i microscope. The complexity of *Quercus ilex* tree rings did not allow to cross-date samples, thus age of recruits must be taken as an approximation due to the possible presence of double or missing rings.

We then separately fitted models for each precipitation level using a power function. We used the nls command (*stats* package, R v 3.5.2; R Core Team, 2019) with starting values of *a*= 1.1 and *b*= 1

$$Y=a D^{b}$$

Where *Y* is the number of tree rings; *D* the average, field measured root-collar diameter; and *a* and *b* parameters to be estimated.

Results:

Figure S4.1: Relationship between age of recruits of *Quercus ilex* and average diameter at the root-collar for both precipitation levels.

Model for the semi-arid

Parameter Estimate Std. Error Pr(>|t|)

a 7.58314 1.43224 <0.0001

b 0.45389 0.05507 <0.0001

Model for the sub-humid

Parameter Estimate Std. Error Pr(>|t|)

a 6.17313 1.39357 <0.0001

b 0.51063 0.06509 <0.0001

Table S4.1: Summary of models relating root-collar diameter of recruits of *Quercus ilex*-measured in the field and age estimated from tree-ring counts at the laboratory of the sampled recruits for each precipitation level.

References:

Gärtner, H., Lucchinetti, S., Schweingruber, FH. (2015). A new sledge microtome to combine wood anatomy and tree-ring ecology. IAWA Journal 36: 452–459.
