# Supplementary material for: Increasing aridity threatens the sexual regeneration of Quercus ilex(holm oak) in Mediterranean ecosystems
Source: PLoS One. 2020 Oct 14;15(10):e0239755. doi: 10.1371/journal.pone.0239755 (PMC7556486; doi:10.1371/journal.pone.0239755)
Supplement: S3 Appendix — (DOCX) [file pone.0239755.s006.docx]

**S3 Appendix. Modelling Logistic hierarchical models (microsite models).**

We start with a logistic regression model that relates the presence or absence of a recruit with the presence or absence of a nurse plant within 50 cm of the sampled point:

$Logit\left( p_{ij} \right)=\beta_{0i}+\beta_{1i}I_{ij}$ [1]

Where $Logit$ is the log of the odds; $p_{ij}$ the probability of finding a recruit in point *j* of plot *i*; $I_{ij}$ an indicator variable which takes the value of 1 if the point is associated with a nurse plant and 0 otherwise; and $\beta_{0i}, \beta_{1i}$are plot-level parameters.

Because the sampling points are clustered in plots, we include a plot random effect for both the intercept and slope parameters:

$\beta_{0i}=\beta_{0}+u_{0i}$

$\beta_{1i}=\beta_{1}+u_{1i}$ [2]

Where $u_{0i}$, $u_{1i}$ are plot level random parameters, assumed to have mean 0 and constant variance, and independent of each other.

After substituting [2] in [1], $\beta_{1}$ is the estimate of the log of the ratio of the odds of finding a recruit in a point associated with a nurse versus one that is not. Therefore, the odds of finding a recruit associated with a nurse plant would be estimated as $exp\left( \beta_{1} \right)$-times the odds of finding a recruit associated with bare soil and no nurse. Only this parameter has a meaningful interpretation, as sampling was retrospective (McCullagh and Nelder, 1989, page 111).

To estimate if the odds ratio depended on plot-level variables, we incorporate those variables in [2] above as follows:

$\beta_{0i}=\beta_{00}+\beta_{01}X_{i}+u_{0i}$

$\beta_{1i}=\beta_{10}+\beta_{11}X_{i}+u_{1i}$ [3]

Where $X_{i}$ is a plot level variable, either continuous (past deforestation intensity, cover of reproductive *Q. ilex*, cover of nurse plants, soil availability, or abundance of herbivores) or categorical (precipitation level).

Substituting [3] in [1] yields:

$Logit\left( p_{ij} \right)=\beta_{00}+\beta_{01}X_{i}+\beta_{10}I_{ij}+\beta_{11}X_{i}I_{ij}+u_{0i}+u_{1i}I_{ij}$ [4]

In this model, $\beta_{11} ,$the parameter associated with the interaction between the plot-level variable and the indicator of the presence of nurse plants at the microsite level, estimates whether the association between recruits and nurse plants presence is modified by the plot level variable under consideration.
